# Systematic Insights into the Relationship between the Microbiota–Gut–Brain Axis and Stroke with the Focus on Tryptophan Metabolism

**DOI:** 10.3390/metabo14080399

**Published:** 2024-07-24

**Authors:** Xinyu Shen, Xiaoqin Mu

**Affiliations:** 1 Genomics Research Center, Key Laboratory of Gut Microbiota and Pharmacogenomics of Heilongjiang Province, College of Pharmacy, Harbin Medical University, Harbin 150081, China; 15543547863@163.com; 2 Translational Medicine Research and Cooperation Center of Northern China, Heilongjiang Academy of Medical Sciences, Harbin 150081, China

**Keywords:** stroke, gut microbiota, gut–brain axis, tryptophan metabolism

## Abstract

Stroke, as a serious cerebral vascular disease with high incidence and high rates of disability and mortality, has limited therapeutic options due to the narrow time window. Compelling evidence has highlighted the significance of the gut microbiota and gut–brain axis as critical regulatory factors affecting stroke. Along the microbiota–gut–brain axis, tryptophan metabolism further acquires increasing attention for its intimate association with central nervous system diseases. For the purpose of exploring the potential role of tryptophan metabolism in stroke and providing systematic insights into the intricate connection of the microbiota–gut–brain axis with the pathological procedure of stroke, this review first summarized the practical relationship between microbiota and stroke by compiling the latest case-control research. Then, the microbiota–gut–brain axis, as well as its interaction with stroke, were comprehensively elucidated on the basis of the basic anatomical structure and physiological function. Based on the crosstalk of microbiota–gut–brain, we further focused on the tryptophan metabolism from the three major metabolic pathways, namely, the kynurenine pathway, serotonin pathway, and microbial pathway, within the axis. Moreover, the effects of tryptophan metabolism on stroke were appreciated and elaborated here, which is scarcely found in other reviews. Hopefully, the systematic illustration of the mechanisms and pathways along the microbiota–gut–brain axis will inspire more translational research from metabolic perspectives, along with more attention paid to tryptophan metabolism as a promising pharmaceutical target in order to reduce the risk of stroke, mitigate the stroke progression, and ameliorate the stroke prognosis.

## 1. Introduction

As an acute cerebrovascular condition, stroke is one of the most important contributors to long-term disability and mortality, affecting millions of people each year worldwide. Ischemic stroke (IS) and hemorrhage stroke (HS) are the two main types of strokes, among which 87% are ischemic, 10% are intracerebral hemorrhage (ICH), and 3% are subarachnoid hemorrhage (SAH) according to the 2024 American Heart Association statistical update [1,2], implying the structural or functional damage to brain tissue caused by the blockage or rupture of cerebral blood vessels. Recent years have witnessed alarming growth as well as the youthful trend of stroke incidence with the prevalence of unhealthy lifestyle and eating habits. In the US, there is a projection that an additional 3.4 million adults over 18 will suffer a stroke by 2030 [1]. Although the therapy of stroke has improved during the past few decades, including intravenous thrombolysis, intra-arterial thrombectomy, recombinant tissue plasminogen activator therapy, and so on, it is limited due to the narrow therapeutic window, individual differences, as well as the potential risk [3]. In addition, dementia, cognitive impairment [4], anxiety, depression [5], and communication disorder [6] can be observed in a large amount of post-stroke cases. Thus, early prevention and intervention of stroke and understanding potential therapeutic targets are particularly essential.

Numerous studies suggested that intestinal disorders accompanied by gut microbial alteration, such as inflammatory bowel disease, irritable bowel syndrome, and constipation, have complex interactions with stroke [7,8,9,10]. Gastrointestinal disorders or gut dysbiosis, on the one hand, often appear as complications in stroke patients; on the other hand, they may increase the risk of stroke [11] or worsen cerebral infarction [12]. The gut environment influences brain function in many ways, involving the central nervous system (CNS), enteric nervous system (ENS), autonomic nervous system (ANS), neuroendocrine system (hypothalamic–pituitary–adrenal axis), and immune system [13]. Likewise, the brain fine-tunes gut activities. The intricate bidirectional communication system between the gastrointestinal tract (GIT) and the brain is called the gut–brain axis (GBA). Since gut microbiota act as an integral regulator in the GBA, the GBA was further extended to the microbiota–gut–brain axis (MGBA) [14].

A key component of the MGBA is tryptophan, an essential amino acid whose metabolism is directly or indirectly regulated by the gut microbiota [15]. Products of tryptophan metabolism, including 5-hydroxytryptamine (serotonin, 5-HT), kynurenines, indole derivatives, etc., exert profound impacts on the pathways related to MGBA [16]. Mounting evidence supports a clear correlation between stroke and Trp metabolism in the MGBA with regard to enzyme activities, metabolite level changes, and related genes [17,18,19]. Promisingly, tryptophan and its metabolites serve as potential biomarkers as well as therapeutic targets of neurological disorders, including stroke, endorsing high precision of diagnosis and alternative treatments.

In this review, we systematically summarize the mechanism of MGBA and comprehensively discuss the role of MGBA in stroke, with a focus on the correlation between tryptophan metabolism and the pathogenesis and progression of stroke, combining the latest knowledge so as to provide a reference for further exploration of the prevention, treatment, and rehabilitation of stroke.

## 2. Microbiota–Gut–Brain Axis and Stroke

### 2.1. Gut Microbiota and Stroke: Epidemiological Relationships

The gut microbiota refers to a community of myriad symbiotic microorganisms that populate the intestinal tract, which harbors over 1000 bacterial species containing more than 100 bacterial phyla [20]. In our bodies, almost 95% of commensal bacteria are dominated by the phyla Firmicutes and Bacteroidetes [21]. As a complex and dynamic metabolic network, the gut microbiota plays a considerable role in the host condition, affecting the immune, inflammation, neural, endocrine, and metabolism processes via GBA.

Emerging evidence elucidated the close relationship between gut dysbiosis and stroke. We looked back on and enumerated 16 case-control studies in the last 5 years (2019–2023) so as to more veritably present such connection (Table A1). By analyzing these case reports, we found that all studies reported differences in gut microbial composition between stroke patients and healthy controls through 16S ribosomal RNA gene sequencing. At the phylum level, the relative abundance of Proteobacteria was increased, while the Firmicutes were mostly reported decreased. At the family level, the amount of Enterobacteriaceae and *Lactobacillaceae* were significantly elevated, while Prevotellaceae were decreased. At the genus level, Lactobacillus, Streptococcus, Akkermansia, and Roseburia were usually upgraded, while Faecalibacterium were reduced. However, the changes in some bacteria, such as Ruminococcaceae, Bacteroidaceae, Coriobacteriaceae, Lachnospiraceae, and Bacteroidetes, remain controversial.

Beyond the altered microbial composition, the gut microbiota itself can be a potential biomarker of the outcome or severity of stroke, verified by the increased Enterobacter, Pyramidobacter, and Lachnospiraceae_UCG_001 in mild ischemic stroke patients, whereas the increased genera, such as Ruminococcaceae_UCG-002 and Christensenellaceae_R-7_group, were found in severe stroke patients [22]. Xu et al. [12] elaborated that Enterobacteriaceae was the most prevalent pathogen in both an IS cohort and mice with middle cerebral artery occlusion (MCAO). In the study of Jiang et al. [23], a positive correlation between Enterobacteriaceae and the severity of IS was found, with more abundance of Enterobacteriaceae, Faecalibacterium, Lachnospiraceae, and Ruminococcaceae but lower lactic acid bacteria of Lactobacillus in the poor outcome group. Furthermore, when inoculating *E. coli*, a common species of Enterobacteriaceae, it turned out that the genes and serum biomarkers associated with immune and inflation systems apparently upgraded, which confirmed that Enterobacteriaceae is a significant influencing factor of stroke [12]. Zheng et al. [24] elucidated that gut microbiota correlated positively with systemic inflammation in cryptogenic stroke (CS) patients and attributed the high level of lipopolysaccharide (LPS) to the elevated amount of Gram-negative bacteria, such as Escherichia–Shigella and Klebsiella of the Enterobacteriaceae family.

The gut microbiota dysbiosis could potentially lead to IS pathogenesis by modulating the host’s traditional risk factors as well [25]; for example, Lactobacillaceae, Enterococcaceae, Streptococcaceae, and Enterobacteriaceae families enriched in IS patients were positively correlated with high-risk factors, including high blood pressure and ApoB/ApoA1 ratio, while negatively correlated with the preventive factor, high-density lipoprotein cholesterol (HDL-C), in the IS patients. Moreover, age, regional, and ethnic variations did make a difference in ischemic stroke when referring to the gut microbiota’s taxonomic changes during the disease.

### 2.2. Mechanisms of the Gut–Brain Axis in Stroke

**Figure 1:**
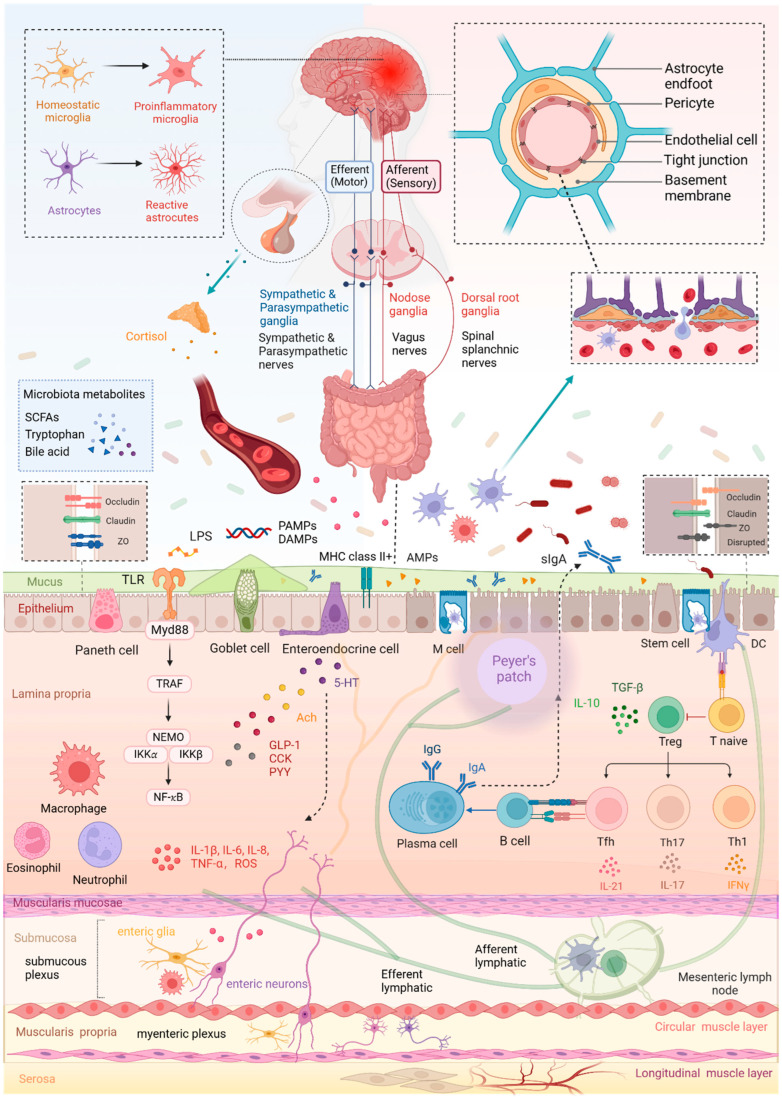
Crosstalk in the microbiota–gut–brain axis associated with stroke. Created with BioRender.com.

In this section, we outline the basic anatomical structure and physiology of the gut–brain axis along with the vital impact of microbiota. On that basis, we have illustrated alterations of the MGBA in stroke to provide a comprehensive understanding of the MGBA as well as its association with stroke. To be specific, this section covers four parts of the microbiota–gut–brain axis, including the barrier structure, defense system, neural network, and humoral communication. Albeit being separately recounted as follows, these parts virtually link together closely and interact with each other in the MGBA (Figure 1).

#### 2.2.1. The Barrier Structure of the Microbiota–Gut–Brain Axis

As the gateway to the communication across the MGBA [26], the barrier of MGBA refers to the specifical cellular interfaces, which principally include the blood–brain barrier and gut epithelial barrier.

- **Gut Barrier and Its Association with Stroke**

The gut barrier can be divided into three layers: (1) The mucus layer, mainly comprising mucins secreted by goblet cells, which are also a nutritional source and adhesion sites for microbiome. (2) The gut epithelial barrier, a selectively semipermeable barrier mainly composed of absorptive enterocytes, enteroendocrine cells, goblet cells, Paneth cells, M cells, and stem cells. (3) The gut vascular barrier, which consists of microvascular endothelial cells attached by fibroblasts, enteric glia, and pericytes. All three layers jointly act as a defense for systemic circulation. To reinforce barrier integrity and restrict paracellular transport, tight junctions formed by transmembrane proteins, including claudins, Occludin, and zonula occludens (ZO), as well as adherens junctions, lie in the gut endothelium [27].

Experimental stroke mice exhibited increased gut permeability, especially vascular permeability in the jejunum and ileum, accompanied by impaired gut mucoprotein, claudin production, and goblet cells. Owing the potential mediation to the upregulated noradrenaline effect in the autonomic nervous system, the study employed β-adrenergic receptor blockers, which significantly ameliorated gut permeability and bacterial dissemination [28,29,30]. A recent study [31] utilizing the MCAO mice model also verified that gut cell death and apoptosis could be reversed by 6-hydroxydopamine, which denervated catecholamine peripheral neurons, and the deficiency of adrenergic receptor ADRB2 alleviated the gut permeability. The upregulated epithelial fructose metabolism gene expression, with hyperplasia of intestinal crypts and goblet cells at 24 h after stroke, was also corroborated, which can be identified as a host compensatory mechanism for the compromised gut barrier. Downregulated tight-junction (TJ) proteins, ZO-1, Occludin, and claudin-1, as well as broken TJ of the intestinal mucosa observed in ischemic stroke mice are simultaneously accompanied by the upregulation of the TNF-α-induced death receptor signaling pathway and inflammation-related proteins NF-κB, iNOS, and MPO, which were suggested to be concerned with intestinal barrier impairment [32].

**Figure 2:**
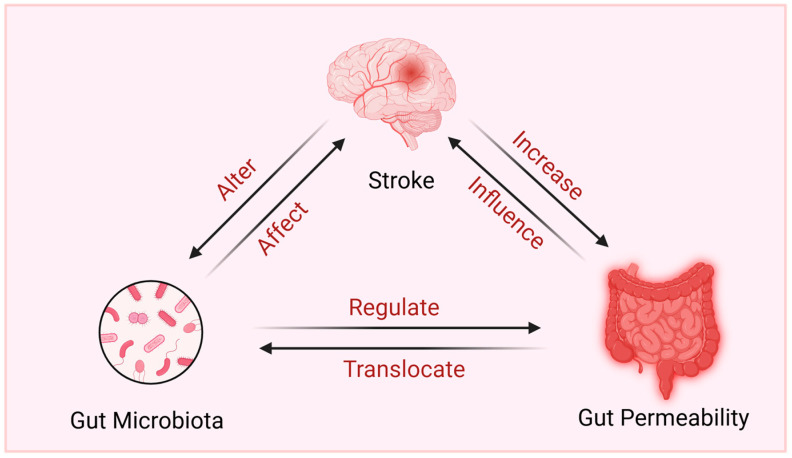
The relationship between stroke, gut microbiota, and gut permeability. Created with BioRender.com.

Gut microbiota can influence the gut barrier by regulating the mucus secretion [33], epithelial cells’ turnover [34], antimicrobial peptides’ production [35], and gut permeability [36]. In that stroke often causes gut dysbiosis, several studies speculated that gut dysbiosis is a cause of damaged gut barrier in stroke, whereas the study of Kumar et al. [31] indicated that stroke-induced alterations of epithelial cells and goblet cells are independent of microbial dysbiosis, as similar changes were also observed in germ-free (GF) mice. Thus, more studies are expected to unravel the specifical role of gut microbial disruption in gut epithelial permeability related to stroke. Furthermore, the disruption of the intestinal barrier may promote translocation of gut microbiota, such as Enterococcus spp., Escherichia coli, and Morganella morganii [28], and increased plasma LBP and LPS, thus exacerbating systemic inflammation after stroke. Taken together, there is an intricate relationship between stroke, gut microbiota, and gut barrier(Figure 2).

- **Blood–Brain Barrier Can Be Influenced by the Gut Microbiota**

Extensively located in capillary vessels of the brain and spinal cord parenchyma, the blood–brain barrier (BBB) mainly comprises capillary endothelial cells along with capillary basement membrane, astrocytes end-foot, pericytes, and extracellular matrix, protecting the brain tissue from the harmful substances in the systemic circulation. Similar to the gut barrier, the BBB has tight junctions as well to maintain its function [37]. Studies have reported that when stroke occurs, cerebral ischemia will induce compromised BBB [38], a key mechanism of brain injury after stroke, which can be caused by oxidative stress, overproduction of free radicals, and iron overload, while the exact molecular mechanisms remain to be explicated.

Previous studies have shown the critical influence of the gut microbiome on the BBB. Germ-free mice exhibited enhanced blood–brain barrier permeability with downgraded tight-junction proteins, Occludin and claudin-5, expression, which can be ameliorated by exposing to the microbiota of pathogen-free mice [39]. Gut dysbiosis mice also displayed decreased TJ proteins in the hippocampus [40] and an impaired cerebral endothelium, as evidenced by three weeks of an antibiotic regimen, which caused endothelial dysfunction in the brain, including an enhanced spontaneous tone as well as blunted L-NAME-mediated constrictions and ATP-mediated dilations, accompanied by lower basal eNOS activity [41]. In another study, the microbiome of spontaneously hypertensive stroke-prone rats disrupted the BBB with enhanced MMP-9 gene expression, and damaged the gut barrier with downregulated E-CAD and Muc-2 gene expression [42]. Under the circumstance of “gut leakiness”, which is similar to a broken gate, it is reasonable that more bacteria and their toxic products’ interactions with cells and neurons, as well as the diffusion to systemic circulation, were allowed, triggering a vast array of immune responses and inflammation with altered pathways along the GMBA. As a result, BBB permeability is affected by the above alteration, directly or indirectly rendering it more accessible to various pathogens. Thus, it can be found in various studies that changes in the gut and brain barriers are positively correlated and concomitant.

Intriguingly, clear similarities can be found in brain and gut barrier structures, which further facilitate the correlation between the gut and brain along the MGBA and implicate the amenability to be regulated by the common signals, including gut microbiota metabolites, for example, SCFAs, especially butyric acid or sodium butyrate, which exert a protective effect on both gut and brain epithelial integrity [43,44], regulating mucus secretion [45] and redistributing ZO-1, Occludin, and claudin protein expression [46,47,48].

#### 2.2.2. The Defense System of the Microbiota–Gut–Brain Axis

- **The Immune System in the Gut**

As the gatekeeper [49], when threatened by a noxious substance, the intestinal immune system, the largest defense system in our body, readily delivers various strong immune responses based on its sophisticated structure [50]. At the mucous layer, molecules, including IgA, and antimicrobial peptides (AMPs) can kill bacteria, inhibit pathogens, and engage in the regulation of immune signals. Other components, such as mucin 2, zymogen granule protein 16, and IgG Fc-binding protein, have also been demonstrated as part of the innate immune mucosal defense or messengers to the host immune system [51]. Right next to the mucous layer is epithelial cells, which are equipped with cell junctions, as illustrated before, further contributing to defend against most pathogens. Beneath the epithelium lies the lamina propria, a noncellular connective tissue, which consists of a large number of immune cells [52], including monocytes or macrophages, neutrophils, eosinophils, basophils, mast cells, innate lymphoid cells (ILCs), natural killer (NK) cells, lymphocytes, and dendritic cells (DCs). These cells act to phagocyte and eliminate microorganisms and secrete anti-inflammatory factors, such as interleukin 10 (IL-10) [53], possessing anti-inflammatory effects to maintain gut homeostasis [54]. During the gut dysbiosis or stroke damage, pathogen-associated molecular patterns (PAMPs), such as LPS, peptidoglycan, and lipoteichoic acids [55], and damage-associated molecular patterns (DAMPs), including ATP, mitochondrial DNA, IL-33, etc. [56,57], can be recognized by the pattern-recognition receptors (PRRs), represented by the Toll-like receptors (TLRs) family, expressing on the surface of enterocytes or other immune cells. TLRs then interact with MyD88 through TIR domains and trigger a cascade, which leads to the translocation of NF-κB into the nucleus, regulating the expression of proinflammatory cytokines, including TNF-α, IL-1, and IL-6, in the innate and adaptive immune mediation [58].

Along with the lamina propria, Peyer’s patches (PPs), mesenteric lymph nodes (MLNs), and isolated lymphoid follicles comprise the gut-associated lymphoid tissues (GALTs), the largest lymphoid in our body, which assumes the primary immune response in the gut [50,59]. Specifically in adaptive immunity, antigen is sampled by antigen-presenting cells (APCs), such as DCs, after entering through microfold cells in the follicle-associated epithelium (FAE) or through the epithelium where MHC class II+ enterocytes may also act as local APCs. Then, the processed antigens are passed on to Peyer’s patches or MLNs to activate CD4+-naive T lymphocytes, which can subsequently differentiate into regulatory T cells or T helper (TH) cells with the expression of integrin α4β7 and the chemokine receptor CCR9, eventually accumulating in the mucosa. B cells mature into plasma cells in the lamina propria, producing IgA to the lumen to bind the antigens, the process of which is influenced by factors such as transforming growth factor-β (TGF-β), IL-10, and signals from T cells as well as DCs [50]. Additionally, some CD8+T cells in lamina propria can be cytotoxic T-lymphocytes (CTL), while some CD4+ and CD8+T cells can be memory cells.

- **The Immune System in the Brain**

In parallel, the responsive immune cells, cytokines, and chemokines, as well as antigens, may also gain access to the bloodstream and enter the brain [60]. After stroke onset, intestinal immune cells, majorly DCs and macrophages, have been found to significantly travel to the central nervous system (CNS), where the trafficking amount is higher than other lymphoid organs [61], which aggravates the brain disease. It was thought that the existence of the BBB and the paucity of specialized antigen-presenting cells render the immune response limited in the CNS [62]. Astrocytes and microglia are two extra types of immune cells, which specifically reside in the CNS parenchyma. Astrocytes are large, ramified glial cells, most abundant in the brain, with the function of immune defense and BBB integrity protection. Microglia, the microphages considered as the earliest cells to sense antigens in the CNS [63], transit to M1/M2 proinflammatory phenotypes [64] and release proinflammatory cytokines under the pathological status, which promote inflammation and, meanwhile, facilitate the interaction with astrocytes.

During the early acute phase after stroke, DAMPs released from damaged cells interact with PRRs to initiate innate immunity, inducing the activation and differentiation of immune cells. Concomitantly, upregulation of adhesion molecules, such as intracellular adhesion molecule 1 (ICAM-1) and p-selectin, contribute to the infiltration of immune cells. The release of inflammatory mediators, including matrix metalloproteinases-9, inducible nitric oxide synthase (iNOS), and reactive oxygen species (ROS), further aggravates cells’ apoptosis and the BBB disruption by damaging tight-junction proteins [65,66]. The damaged blood–brain barrier leaves the entrance open for peripheral intestinal immune cells, such as B cells and T cells, where it was reported that over one-fourth of all invading T cells were from intestinal PPs, resulting in the augmentation of neuroinflammatory responses [67].

As the immune response to stroke is a complex and multistage process [68], innate immunity plays a major role in the early acute stage. During the chronic phase, some immune cells, such as B cells, possess a dual effect in the ischemic stroke progression, with benefits related to the secretion of the anti-inflammatory cytokine IL-10, while adverse effects are mediated by autologous immunoglobulins with respect to chronic inflammation [69]. Cerebral ischemia can induce cell-mediated immunity depression, such as diminished early NK and T cell responses with reduced interferons (IFN) production by Th1 cells, all of which was found to be prevented by blocking the sympathetic nervous system [70]. The stroke-induced immunodeficiency related to spontaneously severe bacterial infections primarily contributes to the morbidity, compared to the direct brain lesions.

- **Gut Microbiota Influences the Immune System**

Moreover, microbiota exert a profound impact on gut and brain immunity along the MGBA during stroke [71]. An improved stroke outcome with increased T and B cells was observed in colonized littermates compared to the GF mice [72]. Relevant alterations in the abundant phyla, such as Actinobacteria, Bacteroidetes, and Firmicutes, will induce T cell priming, including overall increased T helper cells (CD4+) as well as polarized regulatory T cells (Foxp3+), which significantly mediates cerebral protection. Meanwhile, the microbiota influences the level of neutrophil infiltration to affect the ischemic brain. Gut dysbiosis can also cause polarization of proinflammatory Th17 (IL-17+) and Th1 (IFN-1+) cells both in the gut [67] and the ischemic brain, along with the increased Treg cells in the small intestine and less IL-17+ γδ T cells in the meninges. Studies have elucidated that the gut microbiota influences such balance between local intestinal Treg cells and γδT cells by inducing the differentiation of intestinal DCs, ultimately affecting the protective or damaging effects of T cells migrating to the brain after ischemic brain injury. Specifically in the brain, the function and maturation of astrocytes and microglia are regulated by microbiota as well, through the Aryl hydrocarbon receptor (AhR) signaling pathway. As numerous studies have demonstrated the role of the gut microbiota in the development, maturation, and function [73] of peripheral and central immune cells, the influence of dysbiosis under pathological conditions, especially stroke, needs more exploration. Furthermore, metabolites, such as SCFAs, tryptophan (Trp), and bile acid (BA) metabolites, or components, such as LPS and peptidoglycans, of microbiota all significantly influence the immune system [74].

#### 2.2.3. The Neural Network of the Microbiota–Gut–Brain Axis

- **The Enteric Nervous System**

Distinguished from other peripheral organs, the gut possesses an intrinsic complete nervous system, namely the ENS, with extensive regulation of gastrointestinal motility, mucus secretory, gastrointestinal endocrine, epithelial permeability, immunologic, and inflammatory responses [75]. The ENS comprises enteric neurons and enteric glia cells, forming two main plexuses, the submucous (or Meissner’s) plexus between the circular muscle layer and the muscularis mucosa, innervating the muscle motor, and the myenteric (or Auerbach’s) plexus between the longitudinal and circular muscle, responsible for secretory and absorptive control. Neurons of the ENS can be classified by function into intrinsic primary afferent neurons (IPANs), interneurons, motor neurons, and intestinofugal neurons (IFN), or by morphology, mainly into Dogiel type I and type II neurons [76,77]. In general, primary transmitters in enteric neurons include acetylcholine (Ach), serotonin (5-HT), nitric oxide (NO), substance P, vasoactive intestinal peptide (VIP), and calcitonin gene-related peptide (CGRP). The myenteric neurons are mostly cholinergic or nitrergic, while submucosal neurons are mainly cholinergic neurons or VIP-expressing noncholinergic neurons [76]. Beyond the neurotransmitters, enteric neurons as well as enteric glia may also produce cytokines, such as IL-18, colony stimulatory factor 1 (CSF1), and transforming growth factor-β (TNF-β). Therein, IL-18 is necessary for the AMPs production of goblet cells to defend against pathogens, including Salmonella typhimurium [78]. TNF-β also plays an essential role in muscularis macrophages’ development and maintenance, indicating the intimate association between enteric nervous systems and barriers, as well as immune function [79,80].

Loss of enteric neurons has been elucidated in the stroke (pMCAO) model, with central and peripheral galectin-3 release through a TLR4-mediated mechanism involving TAK1 and AMPK [81]. Another study also reported the myenteric neurons’ loss with increased VIP-immunoreactive submucous neurons in the ileum of pMCAO mice, which simulated focal cerebral ischemia, whereas global ischemia models exhibited no significant change, implicating that different types of stroke act differently in enteric neuronal survival [82]. A recent study also uncovered that stroke induced decreased neuronal nitric oxide synthase (nNOS) in enteric myenteric neurons, leading to the injured muscle relaxation and slow gut motility. Furthermore, the impairment can be rescued by denervation of the sympathetic nervous system through 6-hydroxydopamine [83].

- **The Connections between the ENS and CNS**

The ENS is closely linked to the CNS through both afferent and efferent pathways [84]. On afferent pathways, where the gastrointestinal tract (GIT) sends sensory information to the brain, the major extrinsic nerves are the vagus nerve (VN) and spinal splanchnic sensory nerves. The vagus nerve, whose cell bodies lie in the nodose ganglia (NG), which project to the brainstem, predominate the upper gut, such as the esophagus and stomach, while spinal splanchnic sensory nerves, whose cell bodies are in the dorsal root ganglia (DRG), which project to the spinal cord dorsal horn and the dorsal column nuclei, generally fall into sympathetic and pelvic pathways responsible for the lower gut. On efferent pathways where the brain delivers the motor information to GIT, the extrinsic nerves are the sympathetic and the parasympathetic nerves. The sympathetic nerves are adrenergic with inhibitory effects, whereas the parasympathetic nerves are cholinergic with excitatory effects in the gut.

As a mixed nerve, the VN contains four principal components, including general visceral efferent, which is parallel with the GIT-distributed part of parasympathetic fibers; thus, when it refers to GIT, the parasympathetic nerves are synonymous with the VN, which has been profoundly studied. The VN connects with ENS, smooth muscle, and mucosa to innervate the gut, readily detecting various signals, including gut hormones, neurotransmitters, as well as bacterial by-products. The neuroprotective effect of VNs and vagus nerve stimulation (VNS) have been found as a promising intervention in cerebral ischemic stroke. VNS can inhibit pyroptosis with suppressed related inflammatory molecules, such as NLRP3, cleaved caspase-1, and reduced pyroptotic cells and membrane pores. It also inhibited Toll-like receptor 4/nuclear factor kappa-B expression and caused a shift from a proinflammatory phenotype in microglia with reduced NOS and TNF α, which is intricately mediated by the α7 nicotinic acetylcholine receptor (α7nAchR) [85,86].

- **Gut Microbiota Influences the Nervous System**

The gut microbiome sends significant signals in the neural network along the MGBA. Studies found that the depletion of microbiota induced activation of the gut sympathetic ganglia, which can be reversed by colonizing with SCF-producing bacteria [87]. Oral inoculation with the microbiota, such as live *Campylobacter jejuni* (*C. jejuni*), could also lead to the activated neurons bilaterally in the vagal ganglia [88], suggesting that peripheral sensory neurons send an early signal to the brain regarding pathogens or gut microbial alteration. Intrinsically, the microbiota closely interacts with the ENS. Antibiotic (Abx)-induced bacterial depletion led to the loss of enteric neurons in both submucosal and myenteric plexuses in the ileum and proximal colon, as well as reduced enteric glia in the ileal myenteric plexus, which could be recovered by microbial restoration. Furthermore, SCFs were verified to stimulate enteric neurogenesis by restoring enteric glia and neurons [89]. A regulatory circuit may also exist in the microbiome, enteric neurons, and other gut cells, including enteroendocrine cells (EECs) and immune cells. EECs can release ENS neurotransmitters, such as Ach and 5-HT, in response to altered microbiota metabolites [54]. Yan et al. revealed a relationship showing that the microbiota affects the neuronal transcriptome and structure, which subsequently tunes the generation of Treg cells lining the enteric neuron projections in the lamina propria [90]. Beyond the SCFs, tryptophan (Trp), and bile acid (BA), gut microbiota also produce and mediate other neuroactive metabolites, such as glutamate, GABA, Ach, 5-HT, and dopamine, affecting neuronal activities [91].

#### 2.2.4. The Humoral Communication of the Microbiota–Gut–Brain Axis

- **Hypothalamic–Pituitary–Adrenal Axis and Its Association with Stroke**

The hypothalamic–pituitary–adrenal (HPA) axis is one of the principal neuroendocrine pathways and a fundamental part of the MGBA. When exposed to stressful stimuli, such as IL-6 and TNF, triggered by a disease such as stroke, the HPA axis is activated, leading to the production of glucocorticoids or cortisol, the primary stress hormones, which exert an anti-inflammatory and immunosuppressive effect on pathological tissue, along with a negative feedback on the hypothalamus and pituitary glands to control the response [92]. However, chronically elevated levels of glucocorticoids may implicate a pathological condition or underlie a bad prognosis.

A massive response associated with HPA was observed in the early stage of acute stroke, with a continuous high level of cortisol, whereas merely in the initial phase did the concentration of adrenocorticotropic hormone (ACTH) increase [93]. Cortisol levels remained high for at least seven days after stroke, as concluded by a systematic review [94], and exogenous corticosterone contributed to neuronal death with larger infarcts in MCAO mice [95]. It has been reported that post-stroke administration of metyrapone, an inhibitor of glucocorticoid synthesis, decreased the IL-6 level and the infarct size in the ischemic brain [96]. However, glucocorticoid receptor blockade was reported to cause increased blood catecholamine, while the application of prednisolone decreased β2-adrenergic receptor expression on lymphocytes in vitro, possibly reducing the sensitivity to catecholaminergic stimulation following a stroke [97]. ACTH was found to be significantly correlated with the brain lesional volume and functional outcome [93], with the effect of predicting the development of post-stroke depression as a vital and independent biomarker in a three-month follow-up study [98]. Numerous studies have identified a dysregulated HPA axis in stroke patients, and a hyper-activated HPA axis was also demonstrated in diabetic mice after stroke with an augmented inflammatory response [96]. Taken together, these studies show that the HPA axis may be a potential therapeutic target in improving stroke outcomes.

- **Gut Microbiota Influences the HPA Axis**

The gut microbiota is one of important components to activate the HPA axis. A higher HPA axis response to stress was observed in GF mice with plasma ACTH and corticosterone elevation [99]. During the inflammatory condition, bacterial translocation is also able to activate the HPA axis through the disrupted gut and brain barriers. At the genetic level, the microbiota was found to attenuate the expression of Fkbp5, a gene regulating glucocorticoid receptor sensitivity in the pituitary gland, as well as the expression of genes encoding steroidogenesis, such as MC2R and Cyp11a1 in the adrenal gland [100].

- **Gut Hormones**

As important signaling molecules within the GMBA, gut hormones spread throughout the GIT are released by enteroendocrine cells (EECs) to regulate gut motility, appetite, and maintain intestinal homeostasis. Enteroendocrine cells, which only account for 1% of gastrointestinal epithelial cells, secrete various kinds of hormones that act locally on nerve endings, enteric neurons, epithelium, and immune cells or enter the circulation to affect the CNS or other distant sites [101]. There are 12 major types of enteroendocrine cells, including enterochromaffin cells, A cells, and D cells, which secrete more than 20 hormones [102]. The main signals involved in the gut–brain crosstalk are ghrelin, polypeptide YY (PYY), glucagon-like peptide-1 (GLP-1), glucose-dependent insulinotropic polypeptide (GIP), cholecystokinin (CCK), and 5-HT [103]. EECs are equipped with a wide array of sensory receptors, which can detect microbial metabolites, such as SCFAs [104], or bacterial components, such as LPS [105]. These gut hormones may subsequently activate IPANs and extrinsic afferent nerve terminals to influence the functions of the brain [106].

## 3. Tryptophan Metabolism along the Microbiota–Gut–Brain Axis and Stroke

**Figure 3:**
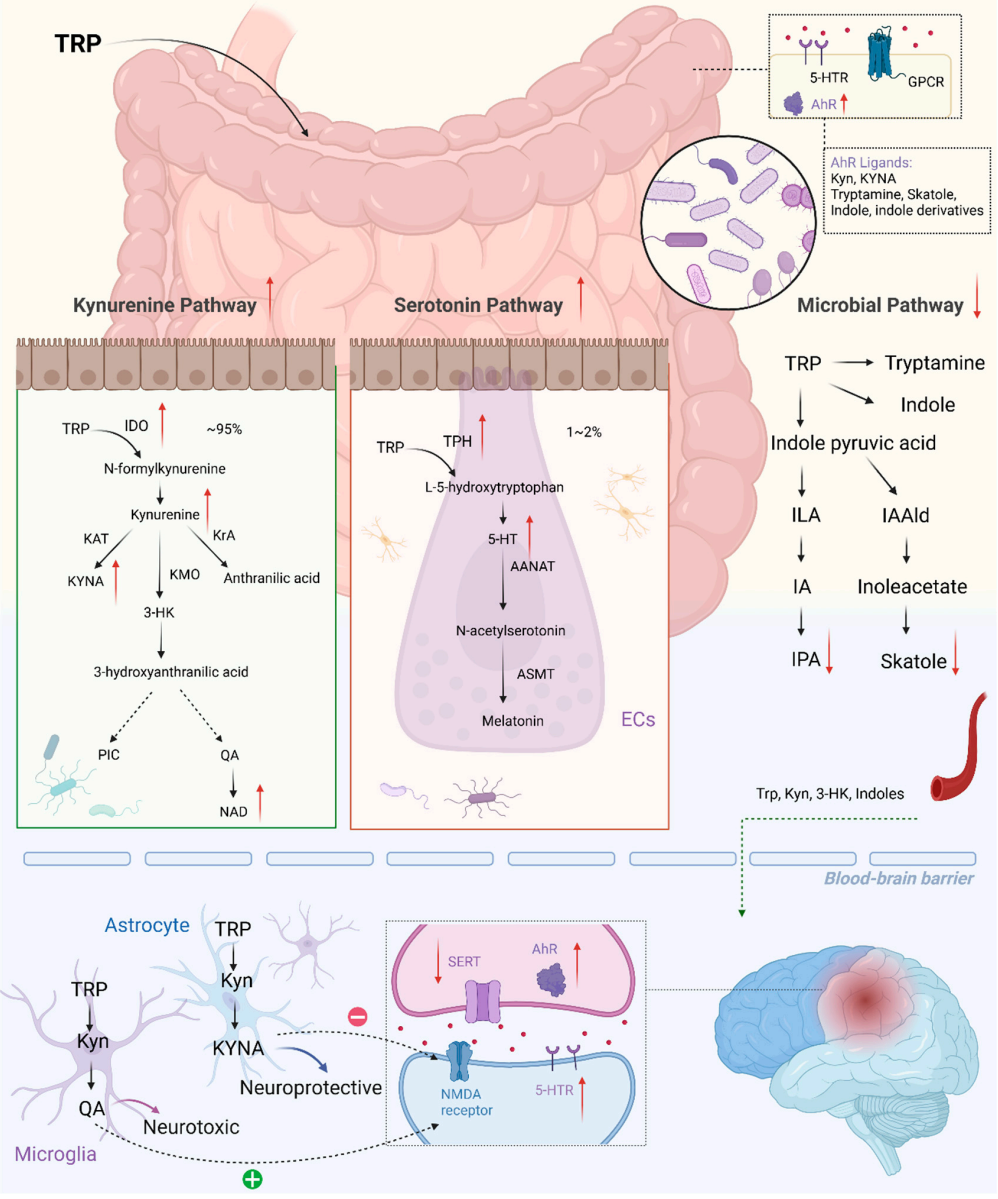
Tryptophan metabolism along the microbiota–gut–brain axis and its main alteration in stroke. Created with BioRender.com.

There is a growing appreciation for tryptophan metabolism along the microbiota–gut–brain axis, which has close associations with stroke. As an essential amino acid, tryptophan mainly originates from the digestion of dietary proteins in the small intestine [107]. Trp is metabolized via three major pathways, with a series of important metabolites in the gut and brain, which experience significant alterations during stroke (Figure 3), exerting a profound effect on the physiological and pathological processes.

### 3.1. Tryptophan Metabolism Pathways along the Microbiota–Gut–Brain Axis

#### 3.1.1. Kynurenine Pathway in the Gut and Brain

The kynurenine pathway (KP) is a key metabolic route that breaks down about 95% of free tryptophan (Trp) [15]. The process is primarily initiated by two crucial rate-limiting enzymes, indoleamine-2,3-dioxygenase (IDO) and tryptophan 2,3-dioxygenase (TDO). IDO is extensively expressed in the body, including the brain and GI tract, with two isoforms, IDO1 and IDO2. IDO1 is pivotal in the immune response and always activated by the inflammatory cytokines, especially interferon (IFN)-γ [108,109], while IDO2 is believed to be basally expressed [110]. TDO, induced by the activation of the HPA axis with increased glucocorticoid, catalyzes 90% of Trp under physiological conditions in the liver and brain [111]. With the aid of the enzyme, Trp is converted to N-formyl kynurenine, which is then catabolized to the pivotal intermediate, kynurenine (Kyn), by formamidase. AhR, a ligand-activated transcription factor that expresses on various cell types, such as T cells, neutrophils, microglia, and astrocytes, plays a master regulatory role in inflammation and immune function with the release of cytokines, including IL-6, IL-17, and IL-22 [112,113]. Kyn, a biologically active metabolite, acts as a ligand for AhR, with the functions of immunity suppression, inflammation, and carcinogenesis [114,115]. Then, Kyn can be further metabolized into three branches, which are kynurenic acid (KYNA) by kynurenine amino-transferase (KAT), 3–hydroxykynurenine (3-HK) by kynurenine-3-monoxygenase (KMO), and anthranilic acid by kynureninase A (KrA).

KYNA, an endogenous neuroprotective agent [116], can activate AhR and G protein-coupled receptor 35 (GPCR35), the activation of which may induce the inositol trisphosphate production and Ca^2+^ mobilization [117]. The 3-HK can influence the immune function by inhibiting the proliferation of CD8+ T cells and suppressing T cell responses [118,119]. Then, 3-HK converts to 3-hydroxyanthranilic acid, whose catabolism leads to the generation of quinolinic acid (QA) and picolinic acid (PIC). The N-methyl D-aspartate (NMDA) receptor is a ligand-gated cation channel, mediating the glutamate signals, with a critical role in the nervous system [120]. Overexcitation of NMDA receptors was suggested to be of great significance to the etiology of brain damage and neurodegenerative disorders, including stroke [121]. Among the major products of tryptophan, KYNA and PIC act as neuroprotective NMDA receptor antagonists, while QA is considered as a neurotoxic NMDA receptor weak agonist with proinflammatory properties [122]. Under pathophysiological conditions, QA can trigger oxidative stress and apoptosis by forming a complex with iron that contributes to ROS in different cells, including neurons and astrocytes, whose decrement, in turn, leads to lower KYNA production, thus enhancing neurological symptoms and chronic inflammation [123]. Oxidative damages to DNA induced by QA were reported to cause the overactivation of poly (ADP-ribose) polymerase (PARP) [124], an enzyme that repairs damaged DNA after oxidative injury, which would further deplete ATP and NAD [125]. Intriguingly, in mammalian cells, de novo from tryptophan is a significant biosynthesis pathway of NAD [126], which can be generated from QA as the end product of the 3-HK branch mediated by the KMO, also a rate-limiting enzyme sensitive to proinflammatory cytokines. During the inflammatory diseases, KP metabolism, as well as the production of NAD, predominantly increase, implicating the enhanced energy demand in the pathological process. Despite that QA and KYNA cannot cross the BBB, Trp, Kyn, and 3-HK can, which contribute to the accumulation and generation of neurotoxic intermediate products, including 3-HK and QA, in the brain, where microglia mainly produce QA, while astrocytes generate KYNA.

#### 3.1.2. Serotonin Pathway in the Gut and Brain

About 1–2% of ingested Trp is metabolized in the serotonin pathway [127], which is initiated by tryptophan hydroxylase (TPH). There are two isoforms of TPH, including TPH1, which expresses in the enterochromaffin cells (ECs), and TPH2 in the CNS and ENS [111,128]. Most 5-HT is produced by enterochromaffin cells in the gut, while the rest is synthesized by neurons, leading to the distinction between mucosal 5-HT, which was related to intestinal inflammation [129], and neuronal 5-HT, which was found to promote intestinal mucosal epithelial growth and turnover, maintain myenteric neuronal density, and influence gastrointestinal motility [130,131]. Trp serves as the exclusive precursor of 5-HT, a primary monoamine neurotransmitter responsible for central neurotransmission, as well as a regulatory hormone in intestinal development and physiological activities [132,133,134,135], depending on the distinct subtypes of 5-HT receptors in various cells and tissues.

The 5-HT receptors, extensively expressed in the CNS, including the hippocampus, hypothalamus, basal ganglia, and cortex [136], are classified into seven principal classes, namely from 5-HT1 to 5-HT7, which all belong to metabotropic G-protein-coupled receptors, except for the ligand-gated ion channel 5-HT3 receptor (5-HT3R) [137]. However, 5-HT3R, which can be found on the terminals of GI vagal afferent neurons, displays an intimate involvement in gut–brain signaling [138]. In response to mechanical or chemical stimulation in the GI tract, 5-HT is released from ECs and acts locally on 5-HT3 receptors present on vagal afferent fiber, triggering smooth-muscle activity during the physiological and pathophysiological processes, such as distention, nausea, and vomiting [138,139]. Highly exposed to luminal contents, 5-HT4R, widely expressed in the intestinal epithelium as well as the ENS, specifically in myenteric neurons [140], is also vital in GI motility and epithelial secretion. Mucosal administration of 5-HT4R agonists promotes mucus secretion by goblet cells and Cl^−^ discharge by enterocytes [141]. Stimulation of 5-HT4R led to anti-proinflammatory, neurogenesis, and neuroprotection responses in the gut, and blocking 5-HT production induced loss of myenteric neurons and inhibition of neuronal differentiation from enteric neuronal stem cells [140]. Moreover, the agonist of the 5-HT1A receptor prevented apoptosis of intestinal epithelial cells and enteric glial cells, with reduced mucosal injury [142], and other 5-HT receptors, such as 5-HT1P and 5-HT2A, have also been verified to mediate different signals within the gut toward the CNS [143]. Additionally, the serotonin-selective reuptake transporter (SERT) exists both in the brain and intestinal epithelium, responsible for 5-HT uptake, effectively clearing serotonin from the synaptic gap and managing 5-HT concentrations [111,139]. Despite that intestinal 5-HT cannot cross the BBB, the free tryptophan that enters the circulation through the gut epithelium may cross the BBB to produce 5-HT in the brain, where some 1% of dietary tryptophan is used for the serotonin biosynthesis [132].

Another well-known product in this branch is melatonin (N-acetyl-5-methoxytryptamine), which is converted from serotonin via N-acetylserotonin through arylalkylamine N-acetyltransferase (AANAT) and acetylserotonin O-methyltransferase (ASMT). Melatonin is a hormone secreted by the pineal gland during the night, with a function in the regulation of circadian rhythm and immunity, as well as an antioxidant property [144]. Dysfunctional serotonin signaling has been widely reported in GI disorders combined with psychiatric symptoms [145].

#### 3.1.3. Microbial Pathway

- **The Gut Microbiota’s Role in Direct Trp Metabolism**

A small amount of Trp can be directly converted by the gut microbiota, primarily into indole and its derivatives. Accumulating evidence suggests that the homeostasis of microbiota–gut–brain interplay plays a central role in the regulation of Trp metabolism. Superior reviews have summarized various gut bacterial species producing different kinds of tryptophan catabolites [146,147]. Overall, phyla Actinobacteria, Firmicutes, Bacteroidetes, Proteobacteria, and Fusobacteria, as well as genera Clostridium, Burkholderia, Streptomyces, Pseudomonas, and Bacillus, are more common and enriched in tryptophan metabolism according to an in silico analysis based on bacterial genomes and gut microbiome data [148]. Specifically, some permanent-resident microbes of the gut, including the genera Clostridium, Bacteroides, as well as Escherichia coli, can metabolize Trp into indole with the expression of tryptophanase (TnA) [149,150]. Indole, viewed as a signaling molecule, affects several bacteria on quantity, virulence, drug resistance, plasmid stability, and biofilm formation, in turn [150]. It also mediates inflammation in innate immune responses [151], modulates the secretion of gut hormones, such as GLP-1, mechanically through inhibiting the voltage-gated K^+^ channel, as well as weakens ATP production with NADH dehydrogenase blockage [152]. Ruminococcus gnavus and Clostridium sporogenes are known to decarboxylate Trp to tryptamine, a β-arylamine neurotransmitter, which induces the release of 5-HT by enteroendocrine cells and binds the 5-HT4 receptor to modulate colonic secretion and promote gut transition [153,154,155]. Along the microbial pathway, indole, tryptamine, skatole, as well as a series of indole derivatives, including indole-3-acid-acetic (IAA), indole-3-lactic acid (ILA), indole acrylic acid (IA), indole propionic acid (IPA), indole-3-acetaldehyde (IAAld), and indoxyl-3-sulfate (I3S), are ligands of AhR [156]. Typically, Bacteroides, Clostridium, Peptostreptococcus, Lactobacillus, and Bifidobacterium are producers of indole-3-lactic acid (ILA), which was suggested to significantly downregulate the expression of IL-8 in gut epithelial cells and suppress NFκB activation in macrophages in a dose-dependent manner via activation of the AhR and nuclear factor erythroid 2-related factor 2 (Nrf2) pathway [157]. Research in human peripheral blood mononuclear cells also exhibited the anti-inflammatory and antioxidative effects, with reduced IL-6 and IL-1b and elevated IL-10 levels under the treatment of IA, which is mainly produced by Peptostreptococcus species [158]. During the inflammation induced by LPS, IPA possessed an inhibitory effect on PI3K/AKT/mTOR signaling pathway. In the study, IPA significantly enhanced the intestinal barrier through increasing claudin-1, Occludin, and ZO-1 levels, as well as facilitating the mucin secretion and the goblet cell function [159].

Indoles are known to cross the BBB and exert a profound effect on the brain [160]. Overproduction of indole in the gut increases the cerebral oxindole level. Supplementation with I3S, IPA, IAld, or tryptophanase in antibiotic-treated mice attenuated CNS inflammation via AhR signaling [161]. Equipped with the potent capacity of scavenging hydroxyl radicals and inhibiting superoxide dismutase, IPA protected primary neurons and neuroblastoma cells from oxidative damage and death induced by Aβ or hydrogen peroxide [162]. Oral administration of IPA mitigated ischemic injury, potentially by reducing DNA damage in pyramidal neurons and lipid peroxidation in ischemic hippocampal homogenates [163]. However, some of the effects can be adverse. For example, intraperitoneally administration of indoxyl sulfate can cause its further accumulation in the prefrontal cortical tissues and cerebrospinal fluid, which leads to behavioral changes and neurodegeneration, including anxiety, depression, and cognitive impairment, accompanied by impaired neuronal cell survival and neural stem cell activity, as well as disturbed expression of serotonin, corticosterone, and brain-derived neurotrophic factor (BDNF) [164].

- **Gut Microbiota Influences the Kynurenine and Serotonin Pathways**

Moreover, the gut microbiota influences the tryptophan metabolism indirectly through the kynurenine and serotonin pathways. Gene expression of the kynurenine pathway enzymes, including kynurenine aminotransferase, tryptophan 2, and 3-dioxygenase in the hippocampus, were changed in GF mice [165]. In GF pigs, tryptophan and kynurenine in the large intestine, as well as 5-HT in most organs, were significantly increased, whereas indole metabolites were generally decreased [166]. A recent study revealed relevant features of KP metabolism in Lactobacillus (L.) reuteri, with the emphasis on the qualitative differences between bacterial and mammalian metabolism. They found that kynurenine readily entered the bacterial cells and was preferentially metabolized to KYNA, whose production was positively correlated with the concentration of kynurenine and bacteria [167]. Beyond the *L. reuteri* from the phylum Firmicutes, bacteria expressing KYN pathways also include Bacteroidetes, Actinobacteria, and Proteobacteria phyla [148]. These microbiota-derived LPS were also found to stimulate colonic immune responses with the upregulation of IDO1 in the Kyn pathway, inducing Trp depletion and Kyn accumulation in the circulation [168]. Under the circumstance of inflammation, the available Trp tend to be shunted into KP rather than serotonin, which can be possibly be explained by the overaction of IDO1 [123]. When administered the antibiotics in combination with a ketogenic diet, downregulation of IDO1 and upregulation of hippocampal kynurenic acid was demonstrated [169]. A dextran sulfate sodium (DSS)-induced colitis mice model also showed increased Kyn, as well as a decreased KYNA level, with a significantly elevated Kyn/Trp ratio, and suggested a correlation with several microbial species, including Bacteroides_acidifaciens, Shigella_sonnei, Parabacteroides_distasonis, Ileibacterium_valens, Bacteroides_vulgatus, and Burkholderiales_bacterium_YL45, which may disrupt the IDO in the cortex, and Parabacteroides_distasonis, Ileibacterium_valens, and Burkholderiales_bacterium_YL45, which were indicated to regulate the KYNA level in serum directly or by affecting KAT2 [170].

Furthermore, gut microbiota metabolites are able to influence the KP as well. In a rat model of bilateral common carotid artery occlusion (BCCAO), SCFA supplementation suppressed the conversion of Trp to Kyn and restored 5-HT levels in the hippocampus and gut, inhibiting the inflammatory augmentation [171]. Butyrate treatment ameliorated serum Kyn accumulation through promoting mitochondrial functions in colonocytes as well as remodeling the gut microbiota in high-fat-diet (HFD) mice [168].

The serotonin pathway is also regulated by the gut microbiota, which plays a key role in promoting 5-HT secretion. Adult GF mice displayed deficient 5-HT concentrations in serum and plasma, with decreased expression of TPH1 in enterochromaffin cells and increased colonic 5-HT transporter SLC6A4, all of which can be reversed by the colonization of indigenous spore-forming microbes (Sp), whose metabolites, such as deoxycholate, a secondary bile acid, also raise 5-HT levels in ECs [172]. In addition, serotonergic neuronal networks were almost absent in GF mice with the alterations of 5-HT4 receptor expression [140]. Activation of 5-HT4R in GF mice may accelerate intestinal transit, concomitant with increased myenteric neurons and innervation of the colonic mucosa [140]. Orally administering gut microbiota from specific pathogen-free mice in GF mice increased 5-HT-positive endocrine cells in the upper GI and colon [173]. Probiotics, such as Bifidobacterium lactis TY-S01, maintained 5-HT in constipated mice with the upregulated mRNA expression of the 5-HT4 receptor [174], and colonization of enteropathogenic Escherichia coli boosted the 5-HT concentration by downregulating the SERT [175]. Besides, frontal cortex 5-HT exhibited negative correlations with the ratio of Firmicutes in total gut microbiota. Treatment with probiotics (fructo- and galacto-oligosaccharide) and prebiotics (Bifidobacterium longum and L. rhamnosus) diminished colonic 5-HT and TPH1 with increased SLC6A4. The decrease in intestinal 5-HT synthesis was then explained for more available tryptophan, possibly converting into 5-HT in the brain through the BBB [176]. Antibiotic-induced gut microbiota intervention was found to significantly increase the 5-HT level and the expression of its receptor 5-HT2A with the upregulation of TPH2 in the hypothalamus [177]. In alcohol-dependent mice, 5-HT content was significantly increased both in intestine and brain tissues after receiving the fecal microbiome from healthy mice [178].

It is believed that BAs activate the bile acid receptor TGR5 on ECs and intrinsic primary afferent neurons to stimulate the release of 5-HT [179], and through the TGR5/TRPA1 pathway, Bacillus subtilis was found to increase 5-HT production from ECs [180]. SFAs, such as butyrate, a positive allosteric modulator of SERT, promoted 5-HT uptake in mouse ileal organoids, and fecal concentrations of both acetate and butyrate were found negatively associated with 5-HT in serum [181]. LPS treatment prenatally increased 5-HT content in serum, the colon, and the medial prefrontal cortex (mPFC) with the increased TPH1, reduced SERT in the colon, and led to a higher serotonin 1B receptor (5-HT1BR) level in the mPFC in autism-like offspring, and they were detected to have lower mRNA expression levels of NAT and ASMT [182].

### 3.2. Tryptophan Metabolism and Stroke

Among the various abnormalities in metabolism induced by stroke, tryptophan metabolism was the most affected, according to an analysis of untargeted metabonomics in rats with stroke [183]. Furthermore, single-nucleotide polymorphisms of the genes encoding the tryptophan metabolism enzymes, such as TPH1, TPH2, KAT1, KAT2, and IDO1, were found to seriously influence the risk and pathogenesis of stroke [17]. Right before or immediately after a stroke, changes in tryptophan metabolism occurred, which were associated with the inflammatory response and oxidative stress.

Therein, the kynurenine pathway was illustrated to be activated right in the hyperacute phase (<4.5 h) of cerebral ischemia [184]. Along the activated kynurenine pathway after stroke, an increased kynurenine:tryptophan ratio and a highly significant decrease in the ratio of 3-hydroxyanthranilic acid:anthranilic acid were previously found correlated with the infarct volume [185]. IDO1 upregulation was always observed in various inflammatory states, including stroke, when the IDO1 mRNA levels increased at an early phase [19]. TDO has gained attention as well. In one study, L-Kyn levels increased in 3 h right after MCAO, with the predominant upregulation of L-Trp as well as TDO, which accounted for L-Kyn biosynthesis in neurons of the ischemic brain. Then, the Kyn activated the AhR very rapidly, which may be causally involved in brain damage at the crucial period when the lesion extended following the ischemic occlusion. Therefore, the inhibition of TDO was found to be a promising therapeutic avenue to inhibit the endogenous apoptotic and anti-neuroprotective pathways in ischemic stroke damage [186]. KYNA was demonstrated to act as a neuroprotective/scavenger/antioxidant factor, which counteracted the tissue damage and neuronal loss induced by stroke. From early phases of stroke, an overexpression of KYNA occurred in mammalian astrocytes exclusively in the infarcted regions and continued at least 21 days post-stroke via the utilization of a highly specific monoclonal antibody against KYN in transient MCAO rats [187]. The negative association between the addition of KYNA and the mortality was found in a multicenter prospective cohort study [188].

AHR is activated and overexpressed in neurons of peri-infarct, ischemic core, as well as the cortex regions in experimental MCAO mice, with a damaging role in stroke physiopathology, as revealed by the AhR agonist, which aggravated the stroke outcome. Haploinsufficiency or total deletion of AhR decreased ischemic damage and were neuroprotective. The cAMP response element (CRE)-binding protein (CREB) is a critical neuronal transcription factor, which mediates the expression of survival and antiapoptotic genes, such as neuroprotective BDNF, against ischemic injury. The increased AhR may inhibit the CREB signaling. Administration of the AhR antagonist TMF boosted the cortical levels of CREB as well as BDNF, and decreased numbers of apoptotic cells in the peri-infarct region of MCAO animals, accompanied by the downregulated proapoptotic proteins p53 and Puma as well as increased antiapoptotic Bcl-X [186]. Likewise, during the ICH, AHR overexpressed primarily in neurons in the peri-hematoma zone, which peaked at 48 h from initial insult. Subsequently, the AHR can recruit RhoA/Bax complexes, playing a detrimental role with respect to mitochondria death and free radical accumulation. Pharmacological application of TMF mitigated ROS accumulation and neuronal apoptosis in the ipsilateral hemisphere, with reduced Bax levels, Mito Sox+ cells, proapoptotic protein cleaved caspase-3, and the biomarkers of damage elicited by oxidized lipids, namely 4-HNE protein. In this setting, the kynurenine pathway was initiated immediately after stroke and kynurenine directly contributed to the AHR activation and substantial oxidative stress, with hampered neurobehavioral performance [189].

Of note, the ligands of AHR can be divided into host-derived kynurenine-based ligands, which are upregulated and detrimental after stroke with worsened microglia survival as well as poor prognosis, and microbiota-derived indole-based ligands, such as IAld and IPA, which decrease especially within the first 24 h in both the plasma and the brain after stroke, along with significant reductions in major bacterial populations involved in the regulation of Trp-derived ligands, such as Bifidobacteriales and Lactobacillales [190]. After stroke onset, activation of microglial AHR by microbial Trp-derived ligands was corroborated to suppress the expression of proinflammatory and neurotoxic genes, including TNF, IL6, IL12A, and NOS2, and boost anti-inflammatory IL10 expression. Moreover, the Trp metabolites also led to increased TGFα and downregulated VEGF-B expression via AHR in microglia, which further influenced the proinflammatory gene expression in primary human astrocytes [191]. Post-stroke treatment in GF mice with the cocktail of IPA and IAld beneficially increased microglial AHR expression associated with a higher surface expression of CD11b, MHC-II, and CD80, ameliorating antigen presentation and co-stimulation immune functions in microglia, which resulted in a reduced infarct size and neurological deficits. All these results showed the two sides of the role of AHR depending on its ligands. Herein, a balanced pool of host-derived and microbiota-derived AHR ligands was highlighted, and via post-stroke administration of indoles, stroke outcomes can be improved [190].

Along the microbial pathway, the serum IPA levels were significantly lower in mice with MCAO. And in patients with coronary artery disease (CAD), IPA was the most downregulated metabolite and decreased more evidently in stroke patients. IPA intragastrical supplementation improved stroke outcomes and the MCAO-induced alterations of the gut microbiome structure, leading to a higher abundance of the probiotic Lactobacillus and Lachnospiraceae_NK4A136_group, which was associated with the enhanced intestinal barrier function and epithelial integrity after IPA treatment. In IPA groups, immune function in MCAO mice was modulated, as proved by the reversing trend of the increased Th17 (CD4+ IL17+) and reduced Treg (CD4+ CD25+ Foxp3+) numbers in the GALT, such as PPs and MLNs, as well as decreasing CD3+ cells and increasing Tregs in the ipsilesional injured brain parenchyma, which further alleviated the neuroinflammation after stroke, with significantly decreased IL1β, IL6, and CCL2 and increased IL10 at both the mRNA and protein levels. The neuroprotective effect of IPA in stroke was further confirmed by the reduction in the activated astrocytes and decreased neuronal apoptosis in the peripheral infarction area. Moreover, the co-cultured experiments in the oxygen–glucose deprivation and reperfusion model revealed that IPA inhibited the A1 phenotype neurotoxic-reactive astrogliosis indirectly via intestinal Tregs [192]. Mechanically, a further study attributed the benefits of IPA to facilitating cholesterol efflux from macrophages to lipid-free apolipoprotein A-I (ApoA-I) through miR-142-5p/ATP-binding cassette transporter A1 (ABCA1) signaling [193]. On the contrary, another study elaborated the hypertensive effect of IPA, and intravenous infusion of IPA in rats increased heart muscle contractility, which can be reflected in the increased stroke volume [194]. In addition, indole-3-lactic acid was found significantly increased at one day after MCAO, but reduced in the chronic phase of stroke, which is potentially related to post-stroke depression [195]. Lower levels of 3-methylindole (skatole) and indoles were discovered in human plasma samples 24 h after IS [196]. In general, higher levels of indole-3-propionate were reported to be correlated with lower all-cause mortality, while indole-3-acetate and indole-3-lactate were positively correlated with fatal cerebral infarction, and indole-3-lactate levels were related to fatal stroke [197].

Higher serotonin levels were associated with the incidence of stroke, including fatal stroke [197]. In the serotonin pathway, TPH1 mRNAs was significantly upregulated in MCAO brains, with increased thrombosis and thromboembolism risk [198], and TPH1 variants were found to be more frequent in heterozygous form in the control group compared to the group of ischemic stroke patients [199], implicating a preventive and therapeutic strategy at the RNA and gene levels. Additionally, an analysis of blood samples collected from AIS patients uncovered that melatonin was significantly increased during the disease, whose synthesis may be provoked by ROS in order to defend against the oxidative injury [200], whereas 5-HT plasma concentrations did not display a distinction [19]. In patients undergoing the hyperacute phase (<4.5 h) of brain ischemia, platelet 5-HT2AR density was higher than in controls, while the platelet density of SERT was significantly lower, which coincides with the analysis of genetic polymorphisms in 834 patients with AIS and TIA, revealing that the presence of the high-expression SERT genotype is linked to a lower risk of ischemic stroke [201]. The patient group also showed temporarily increased urinary 5-HT, which had a role in promoting platelet aggregation, vasoconstriction, and ultimately, a thrombotic process. Nevertheless, these alterations decreased after one day and disappeared at three months from brain ischemia symptoms [184]. Furthermore, 5-HT1AR can also act as a serotonergic target for neuroprotection in cerebral ischemia [202]

## 4. Conclusions

From metabolic perspectives, emerging insights into the impacting role of the gut microbiota and gut–brain axis in cardiovascular diseases shed light on preventing and intervening in the progression of stroke, which offers a promising opportunity for translational research. A variety of effective and accessible intervention strategies have been clinically verified, encompassing probiotics, prebiotics, synbiotics, antibiotics, and diet or fecal microbiota transplantation. To harness the full potential of these interventions, a comprehensive understanding of the core pathways and mechanisms within the microbiota–gut–brain axis is essential. In this review, we systematically summarized the alterations of gut microbiota in stroke and the interactive influence between stroke and the immune, neural, and humoral pathways within the MGBA.

Tryptophan metabolism is a fundamental part of the MGBA, and its intimate correlation with CNS diseases has been widely reported. Herein, based on the profound and comprehensive perception of the MGBA, we further described the tryptophan metabolism in the kynurenine pathway, serotonin pathway, and microbial pathway. We overviewed the advances on the relationship between stroke and tryptophan metabolism, with the expectation of promoting the potentially diagnostic biomarkers and therapeutic avenues from the perspective of tryptophan metabolism along the MGBA, such as targeting the key metabolic enzymes at gene, mRNA, or protein levels, applying the antagonist or agonist of the crucial receptors, and controlling some critical metabolites. Practically, as the tryptophan index has been selected as one of the biomarkers to clarify the close and independent association between dietary patterns and stroke risk, the possibility of regulating dietary habits, including increased intakes of fresh fruits, vegetables, and fish/seafoods, in order to prevent stroke was highlighted [203]. Some specific healthy dietary patterns, such as the plant-based, Mediterranean, and Dietary Approaches to Stop Hypertension (DASH) diet patterns, are associated with elevated IPA levels as well as reduced stroke risk [204]. In addition, compounds extracted from natural products that have effects on tryptophan metabolism may provide profitable and auspicious alternatives for the treatment of stroke with few side effects and high availability [205,206].

On account of the different types and stages of strokes, sample size limitations, regional and ethnic variations, and different designs and durations, discrepancies did exist among the studies, which are expected to be refined later on. In the future, clear and precise mechanisms of gut–brain communications, how the MGBA affects stroke, and more associations between tryptophan metabolism and stroke are to be deeply explored with the utilization of advantageous technologies, such as metabolomics, proteomics, microbiome sequencing, etc. This will prompt advances in the field, shift from association to causation, and open new lines for more possibilities of clinical translatability and application, which will provide better prevention, diagnosis, and treatment of stroke, as well as ameliorative stroke prognosis and recovery.

## Data Availability

No new data were created or analyzed in this study.

## Appendix A

**Table A1:** Major gut microbiota alterations in clinical stroke in case-control studies in the past five years.

Type of Stroke	Cohort Size	α-Diversity in Stroke	Main Changes in Gut Microbiota in Stroke	Year	Refs.
IS	30 stroke patients and 30 controls	No difference in Ace, Chao1, Simpson, and Shannon	↑*Odoribacter*, *Akkermansia*, *Ruminococcaceae e_UCG_005*, *norank_p_Flavobacteriaceae*, *norank_p_Parcubacteria*, *Victivallis*	2019	[22]
			↓*Anaerostipes*, *Ruminiclostridium_5*		
IS	31 stroke patients and 9 controls	No difference in Ace, Chao1, Simpson, Shannon, and observed OTUs	↑*Streptococcus * infantis*, *Prevotella * copri*	2019	[207]
			↓*Blautia obeum*		
IS	79 stroke patients and 98 controls	No difference in Ace, Chao1, Simpson, Shannon, and observed OTUs	↑Proteobacteria *, Actinobacteria *, *Lactobacillus* *, *Lactococcus* *, *Escherichia/Shigella* *, *Streptococcus* *, *Collinsella* *, *Dorea* *	2020	[208]
			↓Firmicutes *, Bacteroidetes *, *Faecalibacterium* *, *Subdoligranulum* *, *Eubacterium* *, *Roseburia* *, *Lachnoclostridium* *, *Butyricicoccus* *		
IS, TIA, and HS	349 stroke patients and 51 controls	↓ Shannon, Simpson, and observed OTUs	↑Proteobacteria, *Escherichia/Shigella*, *Peptoniphilus*, *Ezakiella*, *Enterococcus*	2020	[209]
			↓Firmicutes, Bacteroidetes		
IS	20 stroke patients, 10 post-stroke, and 16 controls	↑Ace, Chao1, and observed OTUs	↑Actinobacteria *, Bacteroidaceae *, Bifidobacteriaceae *, Coriobacteriaceae *, Enterobacteriaceae, Weeksellaceae, Bacillaceae, Paenibaciiaceae, Brucellaceae, Xanthomnadaceae	2020	[210]
			↓Firmicutes *, Lachnospiraceae *, Ruminococcaceae*, Prevotellaceae*		
IS	28 stroke patients and 28 controls	↓Shannon and phylogenetic diversity	↑Enterobacteriaceae, Ruminococcaceae, Veillonellaceae, Lachnospiraceae	2021	[12]
			↓Bacteroidaceae, Prevotellaceae		
AIS	140 stroke patients and 92 controls	N/A	↑Lactobacillaceae *, Enterobacteriaceae *, Porphyromonadaceae *, *Akkermansia* *	2021	[211]
			↓Lachnospiraceae *, *Roseburia* *, *Bacteroides* *, *Faecalibacterium* *, *Blautia* *, *Anaerostipes* *		
AIS and AIS with T2D	150 stroke patients and 55 controls	N/A	↑Enterobacteriaceae *, Proteobacteria, Gammaproteobactera, Deltaproteobacteri, Desulfovibrionaceae, *Dorea* *, *Lactobacillus*, *Megasphaera*	2021	[212]
			↓*Lachnospira* *, *Coprococcus* *, *Prevotella*, *Faecalibacterium*, *Roseburia*		
IS	82 stroke patients and 82 controls	↑Shannon and observed OTUs	↑Lactobacillaceae *, Enterococcaceae *, Streptococcaceae *, Enterobacteriaceae *	2022	[25]
			↓Ruminococcaceae, Lachnospiraceae		
ICH	31 stroke patients and 31 controls	No difference in Chao1, Shannon, Simpson, and observed OTUs	↑Bacteroidetes, *Streptococcus*, *Bifidobacterium*, *Akkermansia*, *Lactobacillus*, *Enterococcus*	2022	[213]
			↓Firmicutes, *Faecalibacterium*, *Clostridium*, *Prevotella*, *Gemmiger*, *Blautia*		
AIS and AIS with PHS	17 stroke patients and 10 controls	↑Chao1 No difference in Ace, Shannon, and Simpson	↑Verrucomicrobia *, Synergistetes *, Actinobacteria, Proteobacteria, *Akkermansia* *, *Olsenella* *, *Escherichia_Shigella*, *Bifidobacterium*, *Ruminococcaceae_UCG_014*	2022	[214]
			↓Firmicutes, Bacteroidetes, *Megamonas **, *Prevotella_9*, *Agathobacter*, *Faecalibacterium*		
CS	30 stroke patients and 33 controls	↑Chao1, Shannon, Ace, and observed OTUs	↑Enterobacteriaceae *, Streptococcaceae *, Lactobacillaceae *, *Escherichia–Shigella* *, *Streptococcus* *, *Lactobacillus* *, *Klebsilla*	2022	[24]
			↓Veillonellaceae *, Faecalibacterium *, Dialister, Roseburia		
HS and IS	20 stroke patients and 20 controls	↓Chao, Ace, Shannon, Simpson, and observed OTUs	↑Fusobacteriota *, Desulfobacterota *, Akkermansiaceae, Fusobacteriota, Ruminococcaceae, Oscillospirales	2023	[215]
			↓Acidobacteriota *, *Bacteroides*		
AIS	90 stroke patients and 60 controls	N/A	↑Actinobacteriota, *Bacteroides*, *Parabacteroides* Faecalibacterium	2023	[23]
			↓Spirochaetes, *Faecallbacterium*, *Prevotella*, *Roseburia*, *Lachnospira*		
IS	10 stroke patients and 21 controls	↓Chao1 No difference in Shannon and Simpson	↑Proteobacteria *, Verrucomicrobiaceae *, Bacteroidaceae *, Streptococcaceae *, Rikenellaceae *, Enterobacteriaceae *, Christensenellacea *, *Phascolarctobacterium*, *Alistipes*, *Sutterella*, *Akkermansia*, *Bacteroides*	2023	[196]
			↓Coriobacteriaceae *, Actinobacteria, Clostridiaceae, Ruminococcaceae, *Anaerostipes* *, *Clostridiales* *		
AIS and AIS with HHTN	200 stroke patients and 90 controls	No difference in Ace and Shannon	↑Streptococcaceae *, Lactobacillaceae *, Marinifilaceae *, *Lactobacillus* *, *Veillonella* *, *Bacteroides*, *Bifidobacterium*, *Klebsiella*	2023	[216]
			↓*Escherichia-Shigella* *, *Streptococcus* *, *Anaerostipes* *, *Butyricicoccus* *, *Blautia*, *Faecalibacterium*		

H-type hypertension (HHTN), Type 2 diabetes (T2D), and phlegm-heat syndrome (PHS). Phylum, Class, Family, *Genus*, and *Species* are presented. * Available *p*-value < 0.05. N/A, no available data.
